# Genome Resource of Raspberry Root Rot Pathogen* Phytophthora gonapodyides*

**DOI:** 10.7150/jgen.94500

**Published:** 2024-04-23

**Authors:** Rishi R. Burlakoti, Sanjib Sapkota, Mark Lubberts

**Affiliations:** 1 Science and Technology Branch, Agassiz Research and Development Centre, Agriculture and Agri-Food Canada, Agassiz, BC, Canada.; 2 Science and Technology Branch, Summerland Research and Development Centre, Agriculture and Agri-Food Canada, Summerland, BC, Canada.; 3 Abbotsford Agriculture Centre, Ministry of Agriculture and Food, Abbotsford, BC, Canada.

**Keywords:** *Phytophthora gonapodyides*, oomycete, Illumina Sequencing, red raspberry

## Abstract

*Phytophthora gonapodyides* is a newly reported oomycetes pathogen associated with root rot of red raspberry. We generated high-quality whole genome resource for *P. gonapodyides*, which was pathogenic on red raspberry. The genome size was 88,717,598 bp with a BUSCO completeness score of 93.9%. This genome resource provides insight on pathogen biology of *Phytophthora* spp. causing root rot of raspberry. To our best knowledge, this is the first complete genome assembly of plant pathogenic *P. gonapodyides*.

## Introduction

Phytophthora root rot and wilt caused by *Phytophthora* spp. is the most serious disease of raspberry (*Rubus idaeus* L.) worldwide, including the Pacific Northwest regions of Canada and USA 1. The pathogen initially infects root of the plant and causes serious damage to entire plants with severe wilting symptoms. *P. rubi* is considered as a major pathogen causing Phytophthora root rot and wilt of raspberry, but multiple species of *Phytophthora* including *P. gonapodyides*,* P. cryptogea*, *P. citricola*, and *P. megasperma* were reported to cause the disease from different regions 1-5. *Phytophthora* spp. survive in infected tissues or soil in the form of mycelia or oospores (sexual spores). When adequate moisture is available on soil, both mycelia and oospores can produce sporangia (asexual spores), which can directly infect root tissue or produce motile spores, zoospores for the infection. *Phytophthora* spp. colonize in plant roots and crown and block water and nutrient movement in the vascular system and ultimately cause root rot, wilting and other symptoms on foliage (yellowing, necrosis, and scorching). High rainfall and average temperatures of 15 to 20°C is favourable for the disease development 1.

Whole genome resources of *P. rubi* strains infecting raspberry in Canada were previously reported and their population diversity was assessed 2. *P. gonapodyides* was reported as a new species of *Phytophthora* causing root rot of raspberry in Canada 3,4. This species was also reported from raspberry in Chile 6. However, the complete genome resource of *P. gonapodyides* infecting raspberry has not been published yet. Here, we report the whole genome resources of *P. gonapodyides*, which is a first step towards understanding molecular mechanisms of pathogenicity and host adaptation of this pathogen. The information can also be useful for managing root rot and wilt of raspberry.

## Materials and Methods

We used the isolate BC14 for the whole genome assembly in this study. The detail of pathogen isolation, culture and DNA extraction methods are described in the previous studies 2-4. The isolate (accession number SRR20227809) was identified as *P. gonapodyides* using multiplex targeted sequencing with degenerate primers of three nuclear genes: heat shock protein90, elongation factor 1 alpha and beta tubulin as described in 3. Genomic DNA of the pathogen was extracted using the MagMAX kit (ThermoFisher Scientific Inc.) following the manufacturer's instructions. For whole genome sequencing, 2 x 150 bp paired-end configuration libraries were constructed using KAPA Hyperprep PCR-free library kit according to the manufacturer's instructions. Sequencing was performed on an Illumina HiSeqX next-generation sequencing device at Admera Health LLC (Plainfield, NJ) according to the manufacturer's directions. We used methods from our previous study 2, to assemble the genome of *P. gonapodyides* isolate BC14. In brief, Spades v3.15 7, was used for the genome assembly and was annotated was performed using Funannotate v1.8.13 (https:// github.com/nextgenusfs/funannotate) with Interproscan 8 and SignalP v6 9 additionally used for functional annotation. Genome completeness was evaluated with BUSCO v5.4.4 using the Eukaryota Odb10 gene set 10.

## Results and Discussion

Illumina sequencing generated a total of 23.7 Gbp of raw sequence data, with a mean coverage of 178x of the *P. gonapodyides* isolate BC14. The detail of genome assembly is provided in Table 1. The assembled genome was 88,717,598 bp in size. The *P. gonapodyides* genome assembled into 29,771 scaffolds and a N50 of 4,047 bp, with a BUSCO completeness score of 93.9%. The total number of predicted genes and proteins were 33,892 and 34,011, respectively. The genome assembly and BUSCO values of *P. gonapodyides* from our study are comparable to previous genome assemblies of *Phytophthora* spp. obtained from short-read and long-read sequences. For example, the genome assembly size and BUSCO values of *P. rubi* recovered from raspberry was 65,960,000 bp with 93.3% completeness 2 and *P. cinnammomi* isolated from avocado was 109,700,000 bp with 97.5% 11. We also compared genome assembly data of *P. gonapodyides* from our study with McGowan *et al.* 12 (Table 1). McGowan *et al.* 12 reported the assembled genome size of 61,088,431 bp and BUSCO value (87.2%) of *P. gonapodyides*, which was isolated by baiting with *Rhododendron* leaves from stream water in Ireland. However, the pathogenicity of the *P. gonapodyides* reported in their study is unknown 12. *P. gonapodyides* isolate from our study was isolated from raspberry plants showing root rot and foliage symptoms and we confirmed the Koch' postulate by inoculating the isolate to the raspberry variety 'Chemainus' 4.

The genome resource of *P. gonapodyides* will be essential for understanding the pathogen biology, developing diagnostic tools for pathogen detection, and exploring potential targets for effective disease management. This genomic data will also be a valuable resource for future comparative genomic and evolutionary studies of plant pathogenic species of *Phytophthora* and other oomycetes.

## Figures and Tables

**Table 1:** Genome assembly statistics of *Phytophthora gonapodyides* isolate BC14 from our study compared with study from McGowan *et al.* 12.

Genome Assembly	*P*.* gonapodyides* Isolate BC14 from our study	*P*.* gonapodyides* from McGowan *et al.* 12
Assembled genome Size (bp)	88,717,598	61,088,431
Number of Scaffolds	29,771	16,449
N50 (bp)	4,047	5455
GC Content	54.59%	55.7%
BUSCO Completeness	93.9%	87.2%
Numbers of predicted genes	33,892	23,348
